# Anoikis-related long non-coding RNA signatures to predict prognosis and small molecular drug response in cervical cancer

**DOI:** 10.3389/fphar.2023.1135626

**Published:** 2023-03-20

**Authors:** Hao Liang, Lan Xiang, Huan Wu, Yang Liu, Wei Tian, Jianhua Zeng

**Affiliations:** ^1^ Department of Obstetrics and Gynecology, The Second Affiliated Hospital, Chongqing Medical University, Chongqing, China; ^2^ Department of Hepatobiliary Surgery, The First Affiliated Hospital, Chongqing Medical University, Chongqing, China; ^3^ Department of General Surgery, The Second Affiliated Hospital of Tianjin, University of Traditional Chinese Medicine, Tianjin, China

**Keywords:** TCGA, cervical cancer, anoikis, LncRNAs, prognosis, immunotherapy, small molecular drug

## Abstract

**Background:** Cervical cancer (CC) is a major health threat to females, and distal metastasis is common in patients with advanced CC. Anoikis is necessary for the development of distal metastases. Understanding the mechanisms associated with anoikis in CC is essential to improve its survival rate.

**Methods:** The expression matrix of long non-coding RNAs (lncRNAs) from cervical squamous cell carcinoma and endocervical adenocarcinoma (CESC) patients was extracted from The Cancer Genome Atlas (TCGA), and highly relevant anoikis-related lncRNAs (ARLs) were identified by the single sample gene set enrichment analysis (ssGSEA) method. ARLs-related molecular subtypes were discerned based on prognosis-related ARLs. ARLs-related prognostic risk score (APR_Score) was calculated and risk model was constructed using LASSO COX and COX models. In addition, we also assessed immune cell activity in the immune microenvironment (TME) for both subtypes and APR_Score groups. A nomogram was utilized for predicting improved clinical outcome. Finally, this study also discussed the potential of ARLs-related signatures in predicting response to immunotherapy and small molecular drugs.

**Results:** Three ARLs-related subtypes were identified from TCGA-CESC (AC1, AC2, and AC3), with AC3 patients having the highest ARG scores, higher angiogenesis scores, and the worst prognosis. AC3 had lower immune cell scores in TME but higher immune checkpoint gene expression and higher potential for immune escape. Next, we constructed a prognostic risk model consisting of 7-ARLs. The APR_Score exhibited a greater robustness as an independent prognostic indicator in predicting prognosis, and the nomogram was a valuable tool for survival prediction. ARLs-related signatures emerged as a potential novel indicator for immunotherapy and small molecular drug selection.

**Conclusion:** We firstly constructed novel ARLs-related signatures capable of predicting prognosis and offered novel ideas for therapy response in CC patients.

## Introduction

Cervical cancer (CC) is a serious health threat to females, and according to WHO data, CC is the fourth leading cause of cancer deaths among females, accounting for 342,000 deaths in 2020 (Sung et al., 2021). The most common histological type of CC is squamous cell carcinoma that accounts for about 75% of all cases, followed by the second most common type of adenocarcinoma that accounts for 20% of all cases (Meng et al., 2021). Unlike other cancers among females, effective measures including effective early screening and HPV vaccination could significantly reduce the incidence of CC and early targeted treatment (Canfell et al., 2020). Current mainstream treatment options for CC patients including systemic surgical treatment and radiotherapy could improve the 5-year survival rate of early CC patients to 91.5% (Bhatla et al., 2018). However, survival outcomes for patients with advanced CC due to postoperative recurrence and distant metastases are unsatisfactory (Marth et al., 2017). Therefore, there was an immediate demand for monitoring biomarkers to evaluate the CC metastasis risk.

Anoikis, a defense mechanism to prevent abnormal cell migration, is mainly manifested as the loss of cell adhesion to ECM and cell apoptosis and death in the process of migration (Paoli et al., 2013). Anoikis plays an essential component in inhibiting tumor cell migration and is important in delaying cancer progression (Adeshakin et al., 2021). Studies have found that tumor cells secrete growth factors and activate EMT signaling pathway to resist Anoikis, which also became a prerequisite for tumor cell metastasis (Kim et al., 2012). Anti-anoikis has become a landmark event in the occurrence of remote cancer metastasis (Kim et al., 2012). However, few studies focused on the correlation between anoikis and distal metastasis of CC. In view of the inability to accurately evaluate the prognosis of advanced CC patients, it was indispensable to explore the mechanism of anoikis in CC to improve its treatment.

Therefore, we developed an assessment system for prognostic risk stratification and constructed an anoikis-related long non-coding RNAs (ARLs)-related prognostic model in CC. We further investigated the relationship among prognostic indicators and immune microenvironment (TME), immunotherapeutic response, and chemotherapeutic drug suitability. The purpose of this study was to formulate a novel prognostic scoring system for CC, which was designed to accurately guide the prognosis of CC and improve its treatment options.

## Materials and methods

### Dataset download and processing

RNA-sequencing data and corresponding clinical information of cervical squamous cell carcinoma and endocervical adenocarcinoma (CESC) samples were available in The Cancer Genome Atlas (TCGA, https://portal.gdc.cancer.gov/) database using TCGA GDC API. We also downloaded mutect2-processed single-nucleotide variants (SNVs) data from TCGA. The expression matrix with TPM format was transformed into log 2 (TPM+1). Samples were filtered using the Sangerbox (http://sangerbox.com/) (Shen et al., 2022) database as follows: 1) removing samples without follow-up information; 2) retaining samples with survival time greater than 0; 3) remove samples without Status. A total of 291 samples were included after screening. RNA annotation file in GENCODE (https://www.gencodegenes.org/) was used to obtain mRNAs and lncRNAs expression matrix. Anoikis-related genes (ARGs) were available in GeneCards (https://www.genecards.org/).

### Recognition of anoikis-related lncRNAs (ARLs) and molecular subtypes

Based on the expression profiles of ARGs, the ARG scores of samples were attained *via* the single sample gene set enrichment analysis (ssGSEA) algorithm (Barbie et al., 2009). ARLs were identified using the rcorr function in the Hmisc package under screening thresholds of |cor|>0.3 and *p* < 0.01 (Hmisc.pdf). Univariate COX models were performed on the ARLs to screen for prognostically relevant ARLs under the threshold of *p* < 0.01. A consistency clustering analysis was carried out on samples in TCGA-CESC to identify molecular subtypes, according to the methods of Wilkerson et al. (Wilkerson and Hayes, 2010).

### Gene set variation analysis (GSVA) and gene mutation landscape in ARLs-related subtypes

For patients in different subtype groups, hallmark gene sets were captured from the Molecular Signatures Database (MSigDB, https://www.gsea-msigdb.org/gsea/msigdb/) and GSVA was conducted using the GSVA package to explore differences in biological pathway variants across groups (Hanzelmann et al., 2013). Next, oncogenic pathway signatures were obtained from Sanchez-Vega et al. (Sanchez-Vega et al., 2018) and differences in oncogenic pathway scores were assessed in subtypes using the ssGSEA method. SNV data from CC were processed in The Genome Analysis Toolkit (GATK) software in the mutect2 plugin. Genes with mutation frequencies ≥3 were screened (fisher test, *p* < 0.05), and the mutation landscape was mapped using the maftools package (Mayakonda et al., 2018).

### Construction of ARLs-related prognostic risk model (APR_Score)

The TCGA-CESC samples were clustered into training and test sets at 1:1 ratio for analysis. In the training set, LASSO and multivariate COX models were executed on prognosis-related ARLs to discriminate ARLs significantly affecting CC prognosis. ARLs-related prognostic risk model was constructed based on the following formula (Simon et al., 2011).

$$APR\_Score=\sum {Expression}_{ARLs}*\beta_{ARLs}$$

In the formula, expression of ARLs represented the expression data of prognosis-related ARLs, and βARLs indicated the COX regression coefficients after normalization. We calculated the ARLs-related prognostic risk score (APR_Score) of each patient in TCGA. Patients were assigned to the high-APR_Score and low-APR_Score groups according to the optimal *p*-value based on survminer package.

### Clinical meaning of APR_Score and association with prognosis

We analyzed the variation in APR_Score and prognosis amongst the molecular subtypes. In this study, for different molecular subtypes and APR_Score groups, K-M curves were evaluated in the training set and the validation set to assess prognostic differences, and ROC curves were used to assess the accuracy of APR_Score. Furthermore, univariate and multivariate COX model analyses were performed on the TCGA-CESC cohort.

### Gene set enrichment analysis (GSEA)

For patients in different APR_Score groups, GSEA was performed to explore differences in the biological pathways involved (*p* < 0.05, FDR<0.25).

### Relationship between CC patients and tumor microenvironment (TME)

For ARLs-related molecular subtypes and APR_Score groups, we quantified the relative abundance of 22 immune cell species in the TME of CC patients using the CIBERSORT algorithm (Chen et al., 2018). Next, we further assessed the immune scores of 10 immune cell species in the TME using the MCP-Count method (Becht et al., 2016). Finally, 47 classes of immune checkpoint genes were obtained from the study by Danilova et al. (Danilova et al., 2019) to assess their expression levels in subtypes of CC patients.

### Construction of nomogram

In this study, we constructed a nomogram for predicting 1-year, 3-year and 5-year survival rates of patients using the APR_Score (rms.pdf), and calibration curves were applied to evaluate the predictive accuracy of the nomogram. Finally, the ROC curves were exploited to validate the clinical use of APR_Score and nomogram.

### Immunotherapy and small molecular drug sensitivity analysis

We assessed TIDE scores in the Tumor Immune Dysfunction and Exclusion (TIDE, http://tide.dfci.harvard.edu/) website between the APR_Score groups. In addition, we computed the half maximal inhibitory concentration (IC50) values of commonly used small molecular drugs in the pRRophetic package (Geeleher et al., 2014).

### Statistical analysis

The packages included in this study were downloaded from R (version 4.1.1, https://www.r-project.org/) and analyzed using R Studio, an integrated development environment (IDE) for the R language. The packages included Hmisc, ConsensusClusterPlus, GSVA, survival, survminer, glmnet, maftools, timeROC, rms, and pRRophetic. Sangerbox was deployed for sample screening and data processing. In this study, *p* < 0.05 was considered statistically significant.

## Results

### Identification of ARLs in TCGA-CESC

**FIGURE 1:**
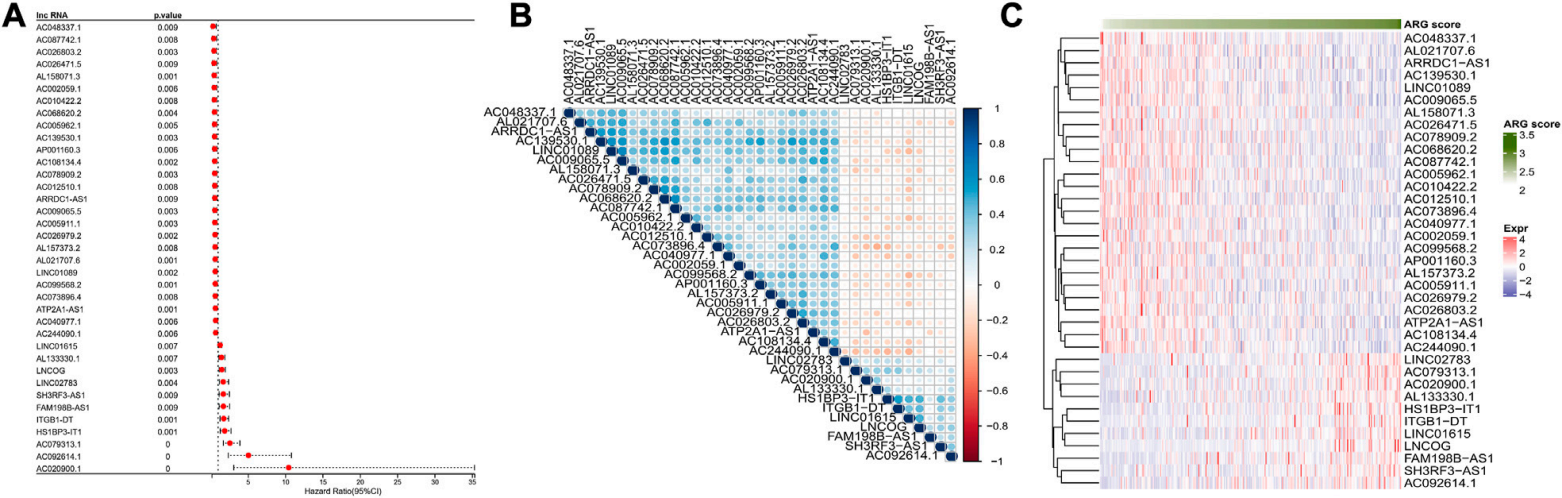
Identification of ARLs TCGA-CESC **(A)** Forest map of ARLs affected OS **(B)** Heatmap of ARLs correlation affected OS **(C)** 37 Expression heatmap OS related ARLs.

Based on annotation file in GENCODE, a total of 14,176 lncRNAs were obtained. Firstly, we computed the ARG score for samples of TCGA-CESC *via* ssGSEA, and 574 ARLs (|cor|>0.3 and *p* < 0.01) were screened based on correlation analysis. This was followed by univariate COX model analysis, filtering a total of 37 prognosis-related ARLs (26 risk factors and 11 protective factors, *p* < 0.01) (Figure 1A). Furthermore, we analyzed the correlation among 37-ARLs, and the correlation heat map was illustrated in Figure 1B. Finally, the expression of 37-ARLs in 291 TCGA-CESC samples was counted, and we observed that 37-ARLs were differentially expressed, showing changes of ARG score in TCGA-CESC patients (Figure 1C).

### ARLs-related molecular subtypes in TCGA-CESC

**FIGURE 2:**
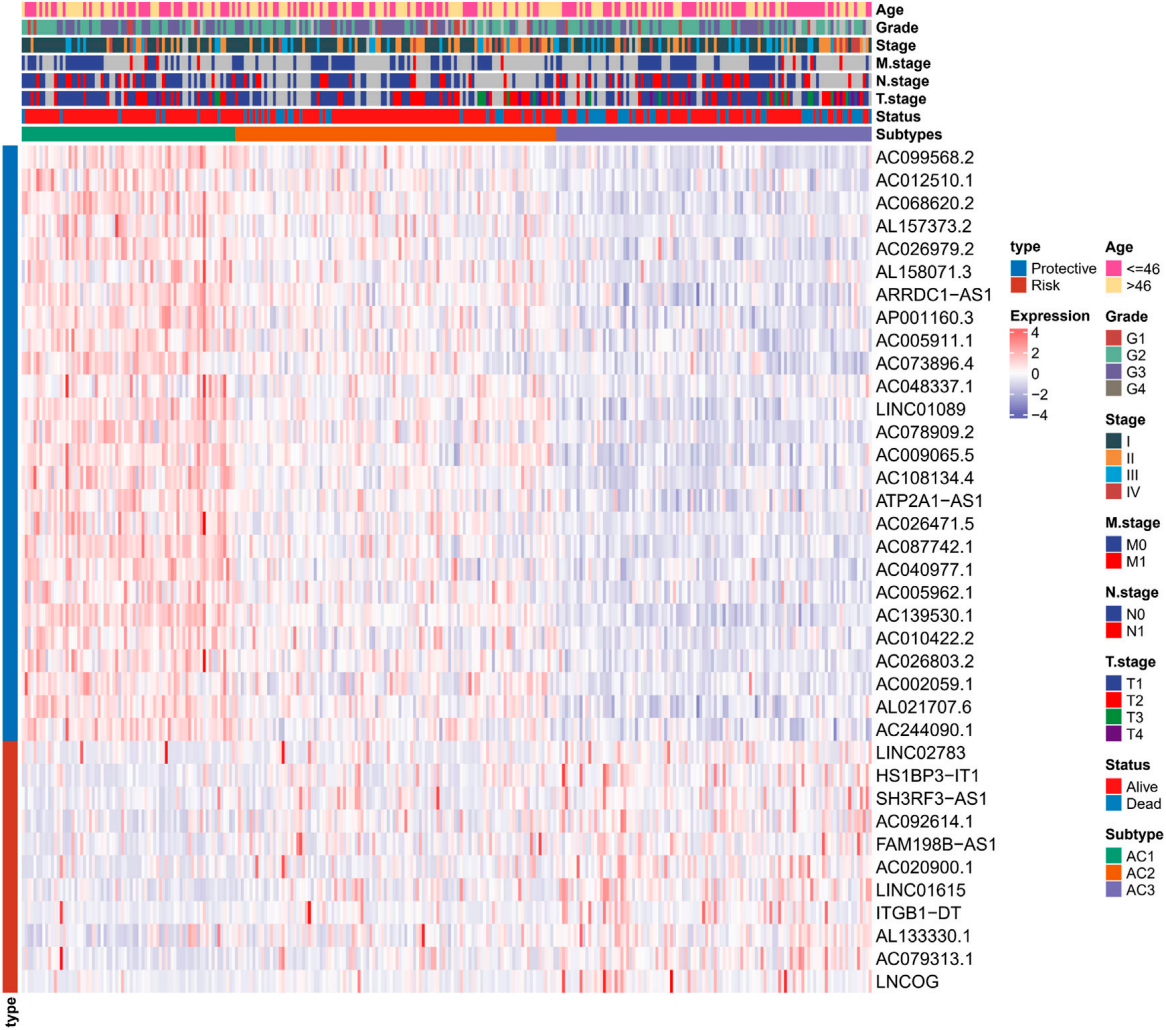
Heat map shows the expression of 37 ARLs in ARLs-related molecular subtypes in CC patients and clinical information.

**FIGURE 3:**
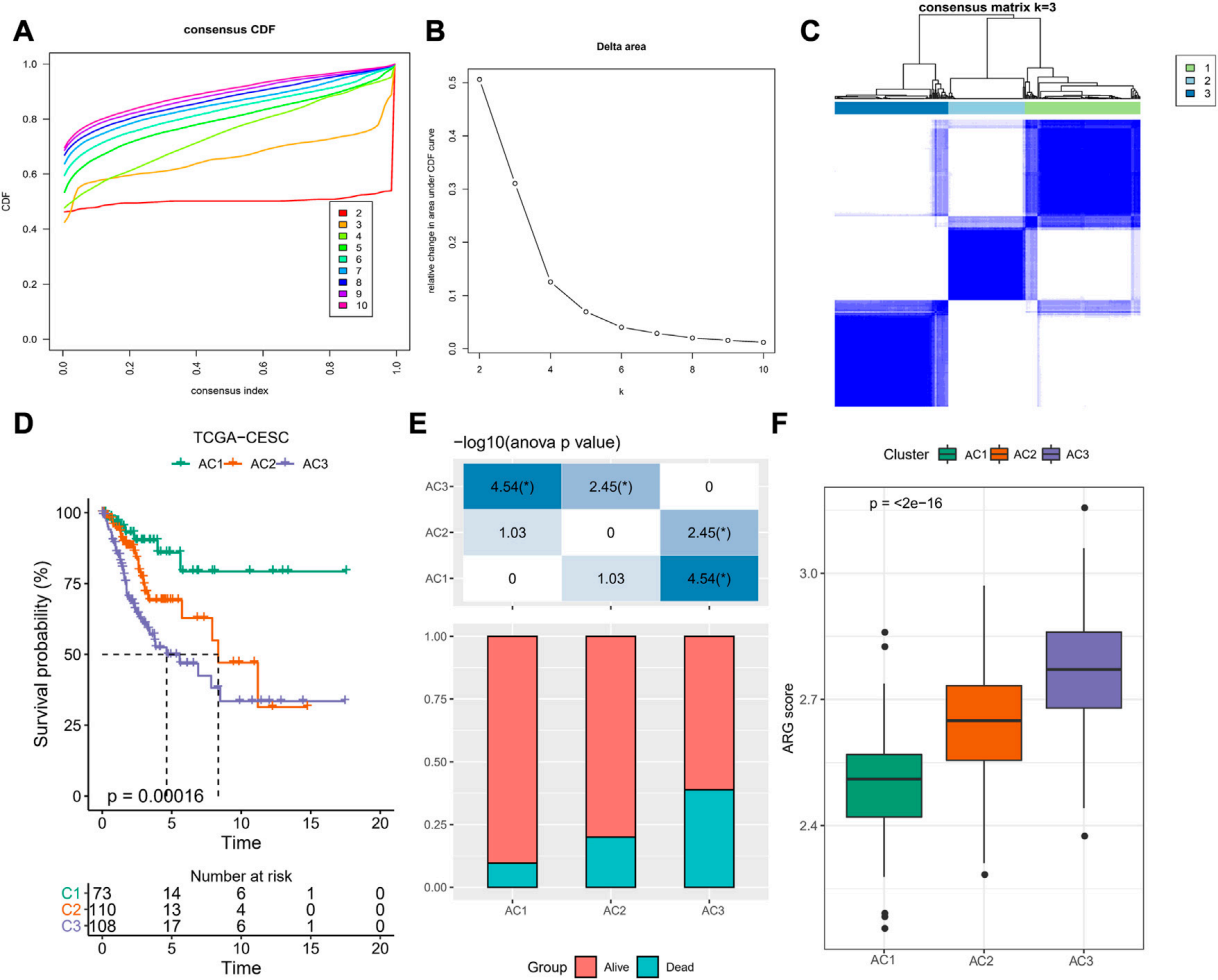
ARLs-related molecular subtypes in TCGA-CESC. **(A)** Cumulative distribution function (CDF) of consensus clustering **(B)** CDF Delta area curve for *k* = 2–10 **(C)** Heatmap of sample clusters shows consensus matrix *k*=3 **(D)** K-M curves of AC1-3 patients in TCGA-CESC **(E)** Statistical graph of patient survival status for patients in AC1-3 **(F)** Distribution of ARG score amongst clusters AC1-3. *p < 0.05.

To determine the potential connections among ARG scores and clinical information, this study counted the expression of clinical information and 37-ARLs in TCGA-CESC patients. We found that risk factors were highly expressed in AC3 and protective factors were highly expressed in AC1 (Figure 2). Classification of TCGA-CESC patients was conducted based on consistent clustering of the 37-ARLs expression matrix. We found that the TCGA-CESC patients could be significantly clustered into three clusters (Figures 3A–C), therefore we derived three ARLs-related molecular subtypes, namely, ARLs-Cluster 1 (AC1), ARLs-Cluster 2 (AC2), and ARLs-Cluster 3 (AC3). Furthermore, the K-M curves showed significant prognostic differences between patients with AC1-3, with AC1 patients having the optimal prognosis and AC3 having the poorest prognosis (Figure 3D). Survival status statistics also showed more deaths among AC3 patients (*p* < 0.05, Figure 3E). We examined the ARG scores of patients in AC1-3 and found that the highest ARG scores were in AC3 patients (Figure 3F).

### Features of TME in AC1-3 subtypes

**FIGURE 4:**
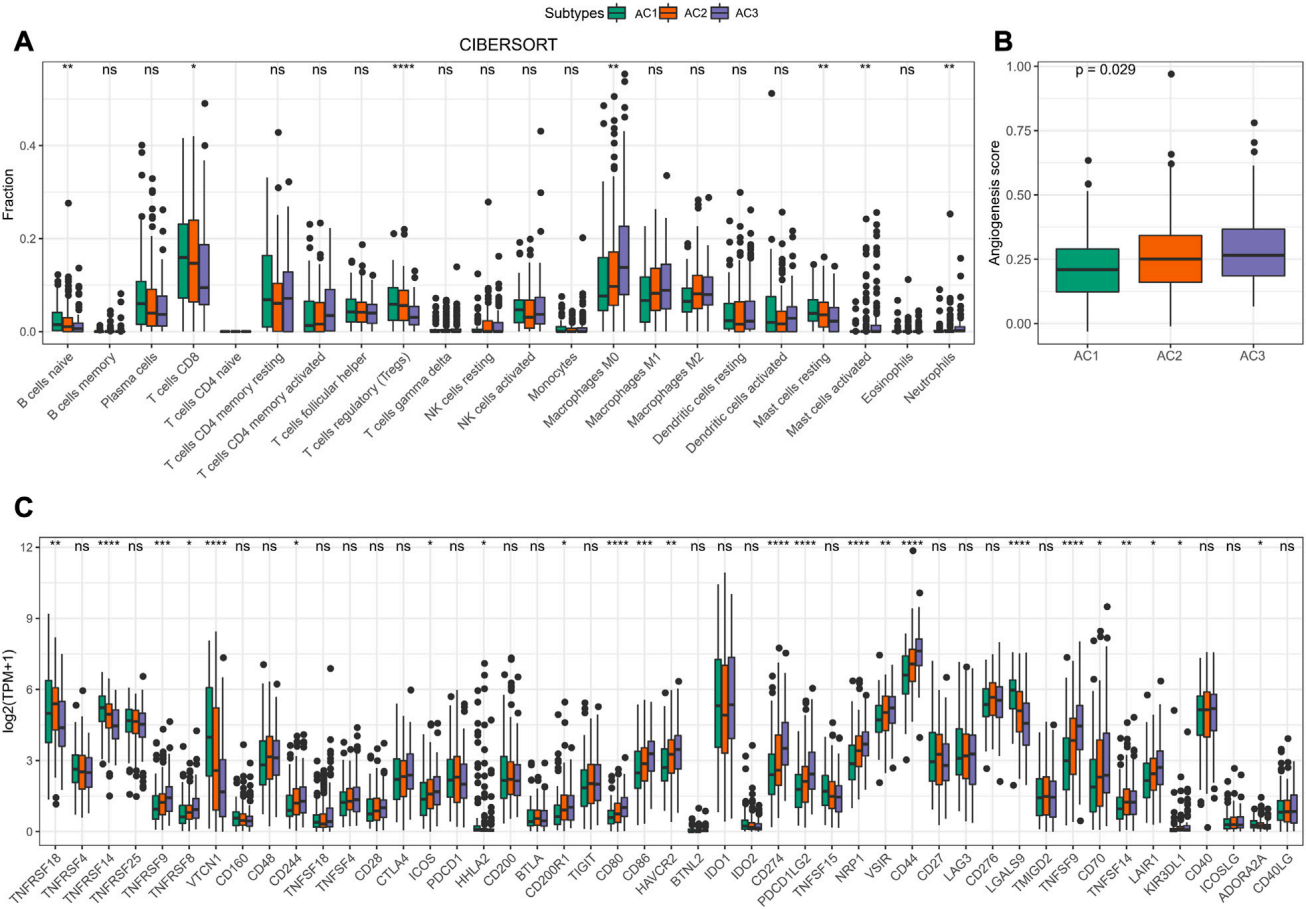
Features of TME in AC1-3 subtypes. **(A)** Distribution of immune cell scores estimated by CIBERSORT amongst three subtypes **(B)** Differences of Angiogenesis scores amongst three subtypes **(C)** Differences of immune checkpoint gene expression amongst three subtypes. ns *p* > 0.05; *p < 0.05; **p < 0.01; *** *p* < 0.001; ****p < 0.0001.

To determine the relationship between ARG score and TME in CC, we assessed the relative infiltration abundance of 22 immune cell species in TME using the CIBERSORT method. We determined that the infiltration abundance of B cells naïve, T cells CD8, T cells regulatory (Tregs), and Mast cells resting was significantly lower in AC3 than in AC1 and AC2. In contrast, the infiltration risk of Macrophages M0, Mast cells activated, and Neutrophils was remarkably higher in AC3 than in AC1 and AC2 (Figure 4A). Accumulating evidence indicated that angiogenesis could influence tumor development and metastasis (Detmar, 2000; Parmar and Apte, 2021). Therefore, we computed the Angiogenesis score in AC1-3 and clearly observed that the poor prognosis AC3 subtype had the highest Angiogenesis score (*p* < 0.05, Figure 4B). In addition, we found that 17 immune checkpoint genes were highly expressed in clusters AC3 (Figure 4C). These results suggested that a higher ARG score was associated with immunosuppressive activity in TME of CC, which could lead to higher Angiogenesis scores, and that the development and metastasis of CC might be inextricably linked to immune escape.

### Genetic landscapes and biological pathways in AC1-3 subtypes

**FIGURE 5:**
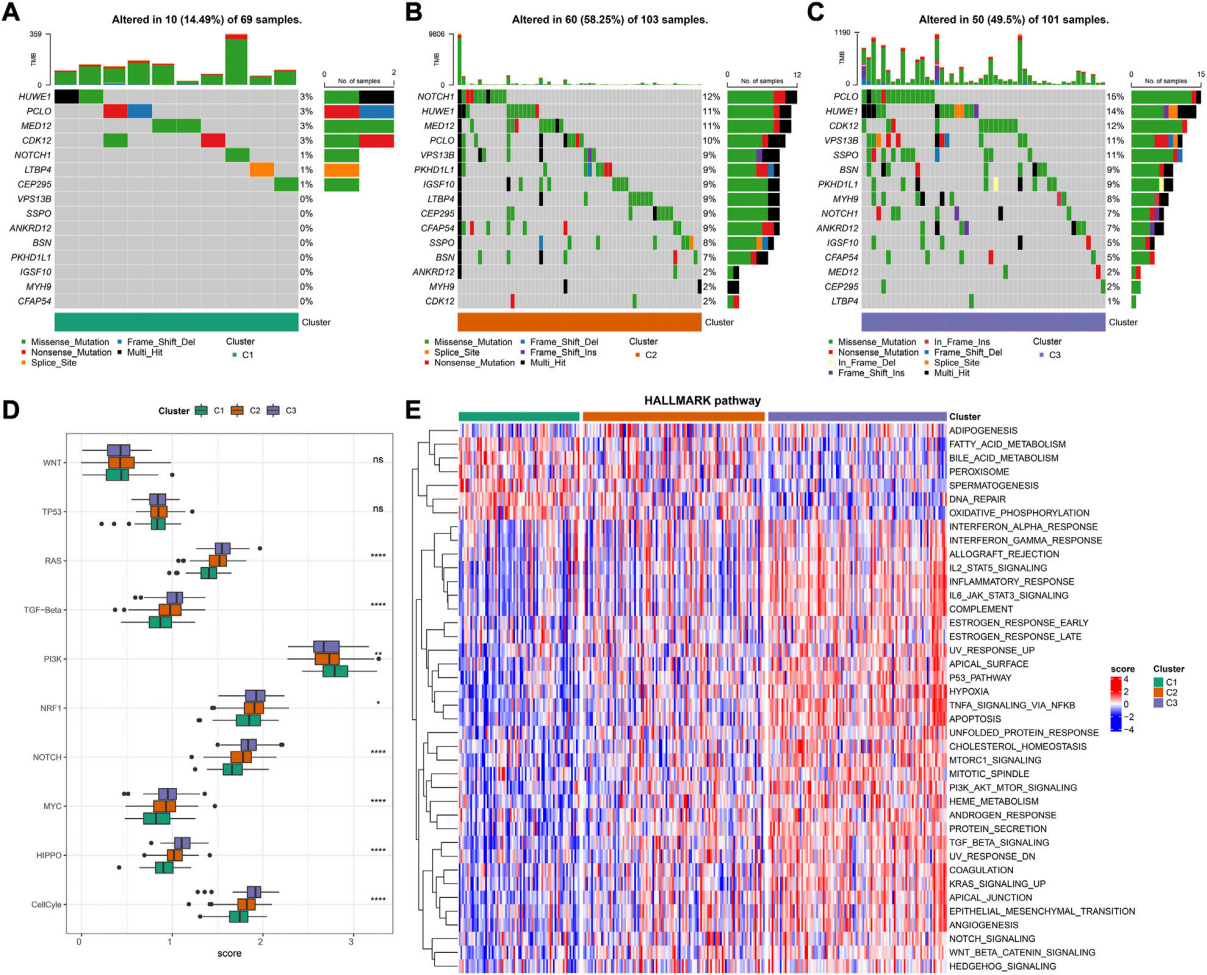
Genetic Landscapes and Biological Pathways in AC1-3 subtypes **(A–C)** The mutation frequencies of top 15 genes in clusters AC1-3 **(D)** Distribution of tumorigenesis-related pathways score in AC1-3 **(E)** Heatmap shows pathway characteristic based on GSVA results. Red represents ssGSEA score is greater than 0 while blue represents ssGSEA score is lower than 0. ns *p* > 0.05; *p < 0.05; **p < 0.01; *** *p* < 0.001; ****p < 0.0001.

Based on the genomic mutational landscape, we found variation among AC1-3 subtypes. AC3 showed markedly higher mutation frequencies than in AC1 and AC2, with the highest mutation frequencies for PCLO, HDWE1 and CDK12 in AC3 (Figures 5A–C). Based on the results of ssGSEA analysis, we noted that AC3 was substantially enriched in tumorigenesis-related pathways containing RAS, TGF-Beta, NRF1, NOTCH, MYC, HIPPO, CellCyle (Figure 5D). Similarly, GSVA results further validated the result because TGF BETA SIGNALING, PI3K AKT MTOR SIGNALING, TNFA SIGNALING VIA NFKB, WNT BETA CATENIN SIGNALING and NOTCH SIGNALING were remarkably activated in AC3 but remarkably inhibited in AC1 (Figure 5E). These results further supported a higher risk of metastasis in patients with CC in AC3, which in turn could lead to the negative prognosis.

### Construction and validation of APR_Score

**FIGURE 6:**
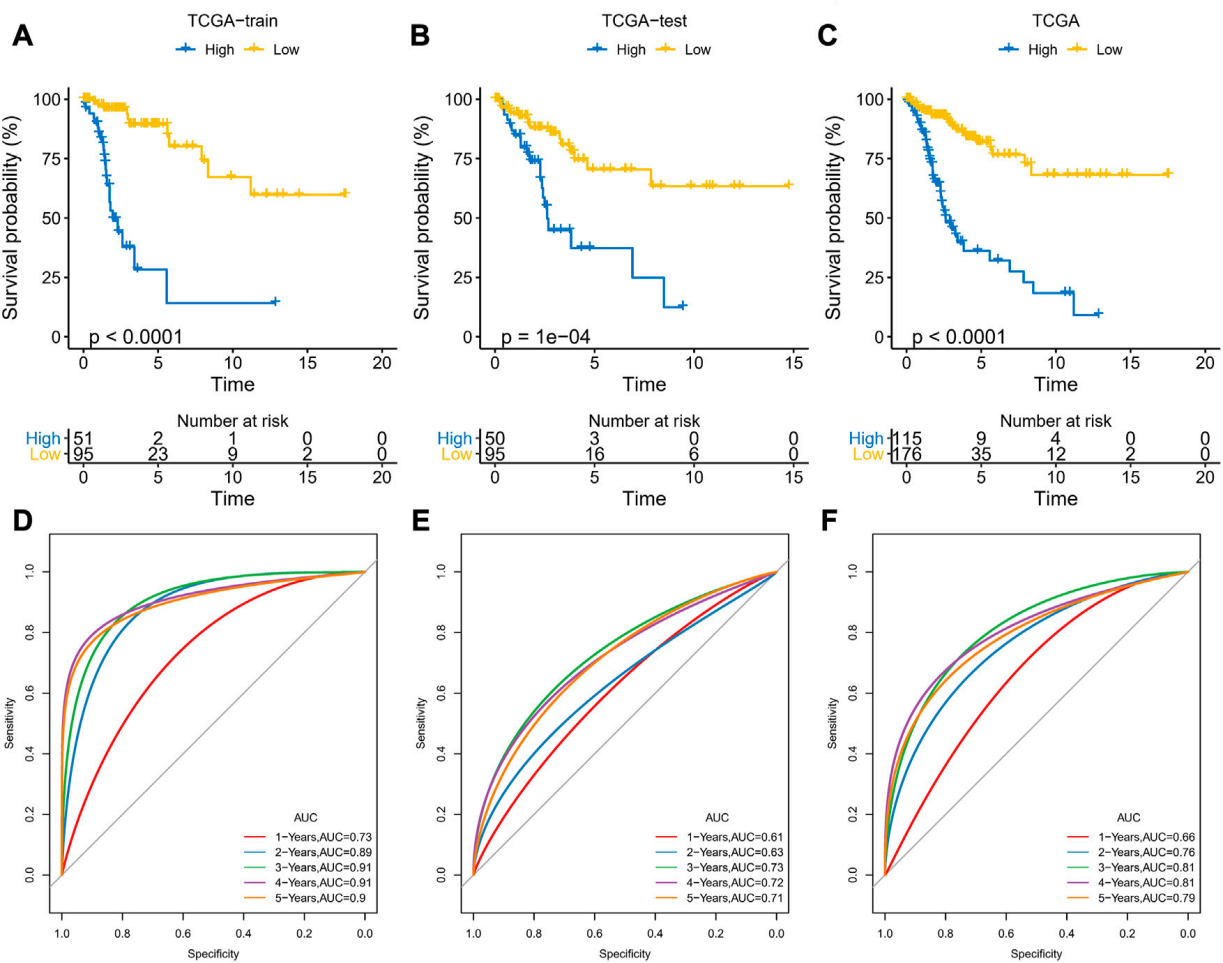
Development and validation of APR_Score **(A–C)** K-M curves for the 7 ARLs in the training set, validation set and TCGA-CESC cohort **(D–F)** ROC curves of the training set, validation set and TCGA-CESC cohort.

The prognostic risk model was constructed based on ARLs by randomly dividing patients in TCGA-CESC into training and validation set at a ratio of 1:1, with the clinical information of the samples in each group shown in Table 1. In the training set, the LASSO COX model was applied to optimize the model, and 11 ARLs were identified based on the penalty parameter lambda and the model trajectory change curve (Supplementary Figure S1). Seven ARLs affecting prognosis were selected based on the multivariate COX model, namely AC092614.1, AL158071.3, AC016394.2, LINC02749, MIR100HG, AC079313.1, and ATP2A1-AS1. According to APR_Score=1.847*AC092614.1+(−0.88*AL158071.3)+(−0.675*AC016394.2)+(−1.099*LINC02749)+0.514*MIR100HG+0.766*AC079313.1+(-0.323* ATP2A1-AS1), the prognostic risk model was assessed. Patients in the training and validation sets were classified into high-APR_Score and low-APR_Score groups by the optimal *p*-value in the survminer. Based on the K-M curves, we noted that high APR_Score scores predicted poorer OS in the training set, validation set and total TCGA-CESC cohort (Figures 6A–C). In the training set, the AUCs were 0.73, 0.89, 0.91, 0.91 and 0.9 for 1, 2, 3, 4 and 5 year(s) of survival, respectively (Figure 6D). While in the validation set and the total TCGA-CESC cohort, APR_Score still showed higher AUC values in predicting 1-, 2-, 3-, 4-, and 5-year OS in CC patients (Figures 6E, F). These results suggested that APR_Score had a high accuracy in predicting OS risk in CC patients and might be a novel prognostic indicator.

**TABLE 1:** Clinical information of CC patients in the training and validation sets.

Characteristics	Train(*N* = 146)	Test (*N* = 145)	Total (*N* = 291)	pvalue	FDR
**Status**				0.76	1
Alive	112 (38.49%)	108 (37.11%)	220(75.60%)		
Dead	34 (11.68%)	37 (12.71%)	71 (24.40%)		
**T.stage**				0.49	1
T1	70 (24.05%)	67 (23.02%)	137 (47.08%)		
T2	30 (10.31%)	37 (12.71%)	67 (23.02%)		
T3	9 (3.09%)	7(2.41%)	16(5.50%)		
T4	3 (1.03%)	7 (2.41%)	10 (3.44%)		
Uknown	34 (11.68%)	27 (9.28%)	61 (20.96%)		
**N.stage**				0.91	1
N0	63 (21.65%)	65 (22.34%)	128 (43.99%)		
N1	29 (9.97%)	26 (8.93%)	55 (18.90%)		
Uknown	54 (18.56%)	54 (18.56%)	108 (37.11%)		
**M.stage**				0.99	1
M0	53 (18.21%)	54 (18.56%)	107 (36.77%)		
M1	5 (1.72%)	5 (1.72%)	10 (3.44%)		
Uknown	88 (30.24%)	86 (29.55%)	174 (59.79%)		
**Stage**				0.77	1
I	84 (28.87%)	75(25.77%)	159 (54.64%)		
II	29 (9.97%)	35 (12.03%)	64 (21.99%)		
III	20 (6.87%)	21 (7.22%)	41 (14.09%)		
IV	11 (3.78%)	10 (3.44%)	21 (7.22%)		
Uknown	2 (0.69%)	4 (1.37%)	6 (2.06%)		
**Grade**				0.86	1
G1	8 (2.75%)	10 (3.44%)	18 (6.19%)		
G2	65 (22.34%)	64 (21.99%)	129 (44.33%)		
G3	59 (20.27%)	57 (19.59%)	116 (39.86%)		
G4	0 (0.0e+0%)	1 (0.34%)	1 (0.34%)		
Uknown	14 (4.81%)	13 (4.47%)	27 (9.28%)		
**Age1**				0.86	1
<=46	76 (26.12%)	73 (25.09%)	149 (51.20%)		
>46	70 (24.05%)	72 (24.74%)	142 (48.80%)		

### Correlation between APR_Score and clinical characteristics

**FIGURE 7:**
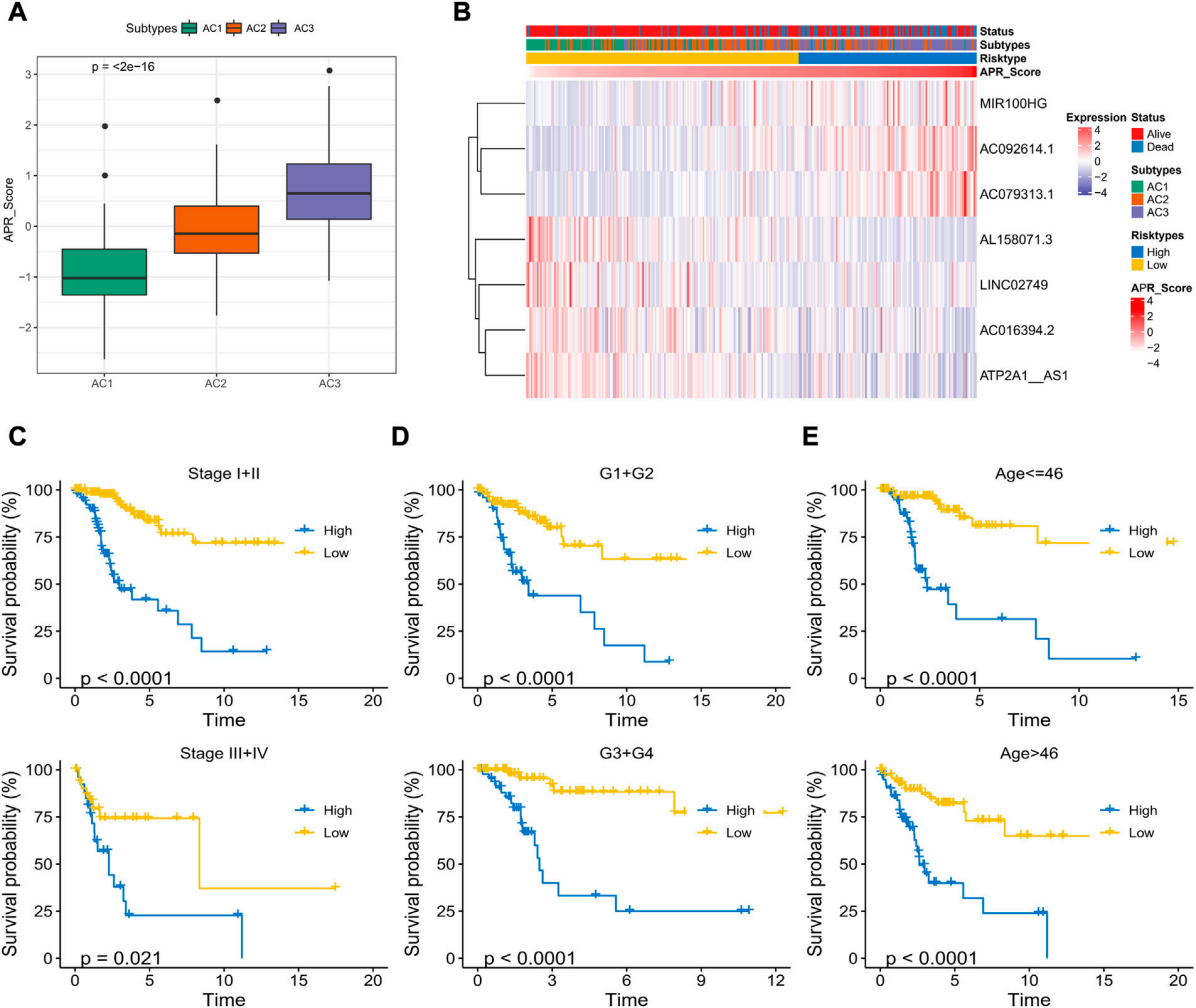
Correlation between APR_Score and clinical characteristics of CC **(A)** APR_Score in AC1-3 subtypes **(B)** Heatmap showing the expression of 7 OS-related ARLs in ARLs-related molecular subtypes and APR_Score groupings **(C–E)** K-M curves for patients with high APR_Score and low APR_Score in Stage, Grade, and Age subgroups.

To determine the relationship between APR_Score and clinical characteristics, this study discussed the interaction between APR_Score and different clinical features. First, we found that the APR_Score was the highest in the poor prognosis AC3 subtype, with more death cases in the high APR_Score group (Figures 7A, B). Expression differences of AC092614.1, AL158071., AC016394.2, LINC02749, MIR100HG, AC079313.1, ATP2A1- AS1 in different types of patients are shown in Figure 7B. In addition, to compare the meaning of APR_Score in clinicopathological subgroups for CC prognosis, K-M survival analysis was performed on patients with high APR_Score and low APR_Score in clinicopathological subgroups. The results showed that patients with high APR_Score had markedly poorer OS than those with low APR_Score in Stage, Grade and Age subgroups (*p* < 0.0001, Figures 7C–E). Additionally, we compared the differences of clinical characteristics (N satge and M stage) between risk groups in TCGA-CESC cohort. The patients with metastasis (including lymph node metastasis and distal metastasis) in the high-APR_Score group were more than those in the low-APR_Score group (Supplementary Figure S2).

### Nomogram for predicting survival rate of CC

**FIGURE 8:**
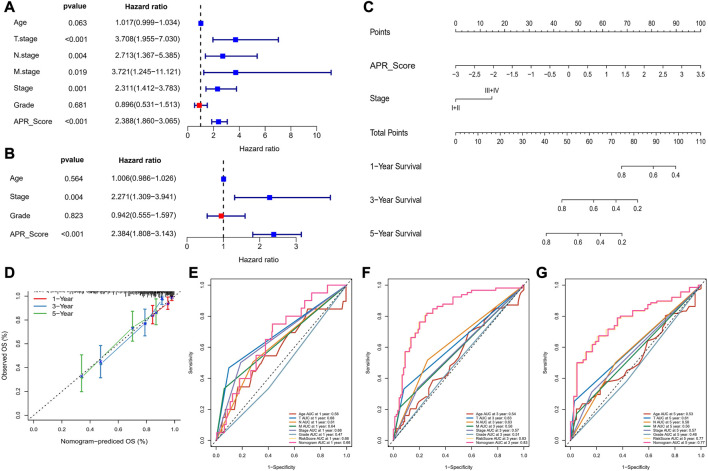
Nomogram for predicting survival rate of CC **(A, B)** Forest plots for univariate and multivariate COX models for APR_Score **(C)** Nomogram of APR_Score and Stage **(D)** Calibration curves for 1-year, 3-years and 5-years survival **(E–G)** The ROC curves of a variety of clinical features for overall OS at 1-year, 3-year and 5-year.

To further discuss the clinical value of APR_Score in CC patients, univariate and multivariate COX model analyses were performed, and we determined that APR_Score and Stage were clinically significant for CC prognosis (*p* < 0.05, Figures 8A, B). Due to a close correlation between APR_Score and Stage and prognosis, we created a nomogram based on APR_Score and Stage to assess OS of CC patients (Figure 8C). The calibration curves showed a great overlap between Nomogram predicted survival at 1-, 3- and 5-year OS with actual observations, indicating that nomogram was a reliable tool for predicting OS (Figure 8D). We also found that the nomogram and APR_Score showed higher accuracy in predicting 1-year, 3-year and 5-year OS when compared to Age, TNM Stage, Stage and Grade (Figures 8E–G).

### TME activity assessment in APR_Score groups

**FIGURE 9:**
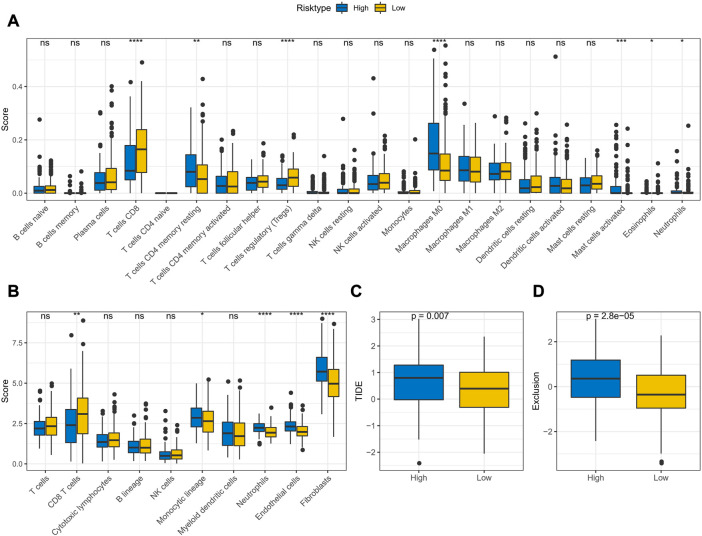
TME activity assessment in APR_Score groups **(A)** Results of immune cell scores estimated by CIBERSORT between APR_Score groups in TCGA-CESC cohort **(B)** Results of immune cell scores using MCP-count between APR_Score groups in TCGA-CESC cohort **(C)** Distribution of TIDE score between APR_Score groups in TCGA-CESC cohort **(D)** Exclusion scores in high-APR_Score group and low-APR_Score group. ns *p* > 0.05; *p < 0.05; **p < 0.01; *** *p* < 0.001; ****p < 0.0001.

The CIBERSORT and MCP-count algorithms were applied to assess the abundance of immune cell infiltration and immune score in TME of APR_Score groups. CIBERSORT results, as presented in Figure 9A, showed that T cells CD8, Tregs infiltration abundance was lower in high-APR_Score group. MCP-count results also showed lower immune score of CD8 T cells in high-APR_Score group (Figure 9B). Then, to discuss the potential connection between APR_Score and immunotherapy response, we assessed TIDE scores and Exclusion scores in the APR_Score groups. TIDE scores and Exclusion scores were slightly higher in the high APR_Score group than in the low APR_Score group, suggesting that patients with high APR_Score were more likely to experience immune escape and less responsive to immunotherapy (Figures 9C, D).

### Association between APR_Score and chemotherapy drug sensitivity

**FIGURE 10:**
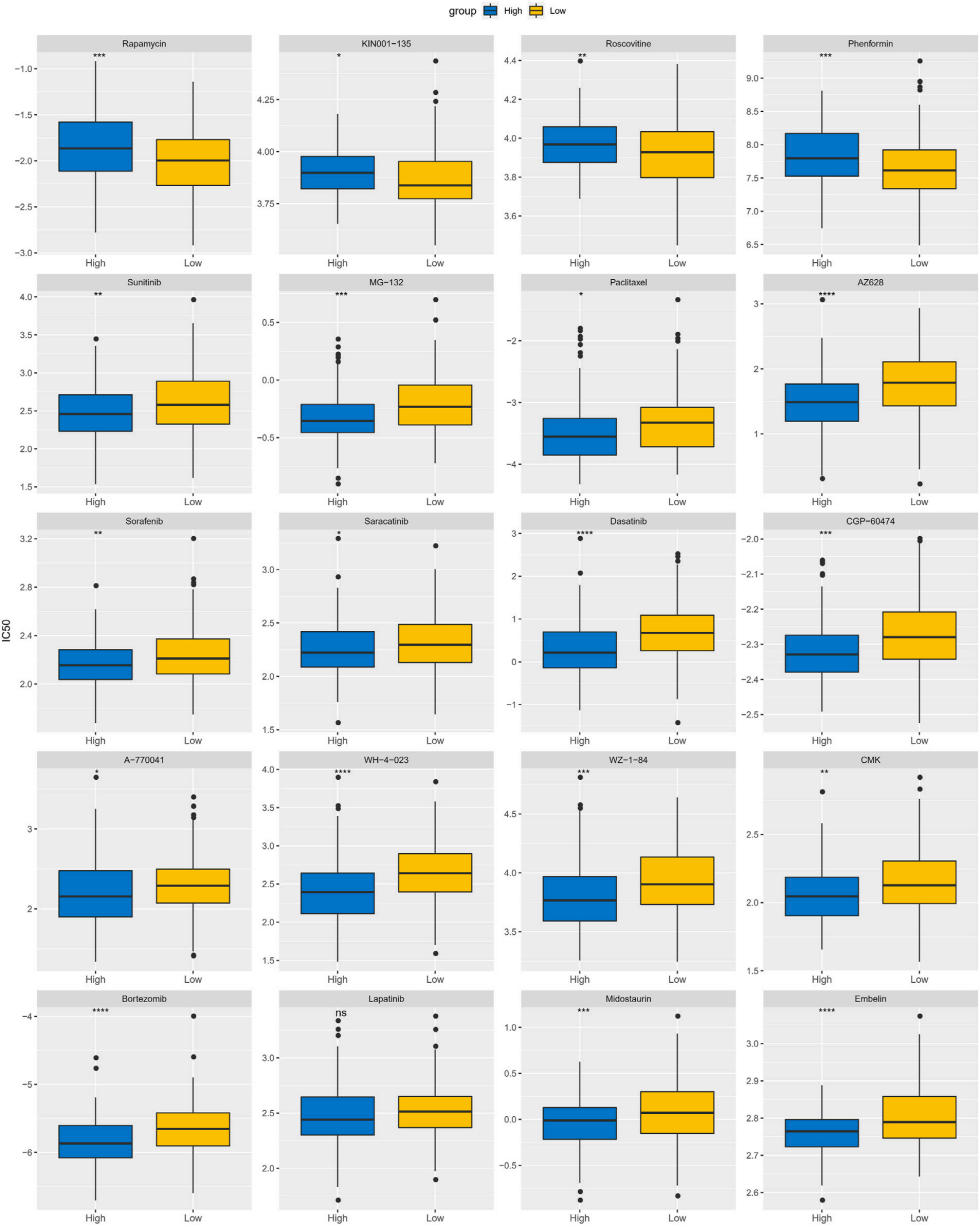
Differences of IC50 values for 20 chemotherapy drugs between APR_Score groups in TCGA-CESC cohort. ns *p* > 0.05; *p < 0.05; **p < 0.01; *** *p* < 0.001; ****p < 0.0001.

Until immunotherapy was proposed as an alternative treatment for CC, conventional resection and radiotherapy were the dominant treatments (Serkies and Jassem, 2018). In this study, to discuss the potential of APR_Score as therapeutic response marker for predicting chemotherapeutic agents, we evaluated the IC50 values of 20 chemotherapeutic agents in TCGA-CESC. Initially, correlations between APR_Score and drug IC50 values were computed, and highly correlated drugs were selected for comparison in the APR_Score groups (Supplementary Figure S3). We found positive responses to small molecular drugs including Rapamycin, KIN001-135, Roscovitine, Phenformin treatments in the low APR_Score group, while the high APR_Score group responded positively to Sunitinib, MG-132, Paclitaxel, AZ628, Sorafenib, Saracatinib, Dasatinib, CGP-60474, A-770041, WH-4-023, WZ-1 -84, CMK, Bortezomib, Lapatinib, Midostaurin, Embelin treatments (Figure 10). Overall, the APR_Score was correlated with the sensitivity to small molecular drugs.

### Biological pathway characterization of APR_Score groups

**FIGURE 11:**
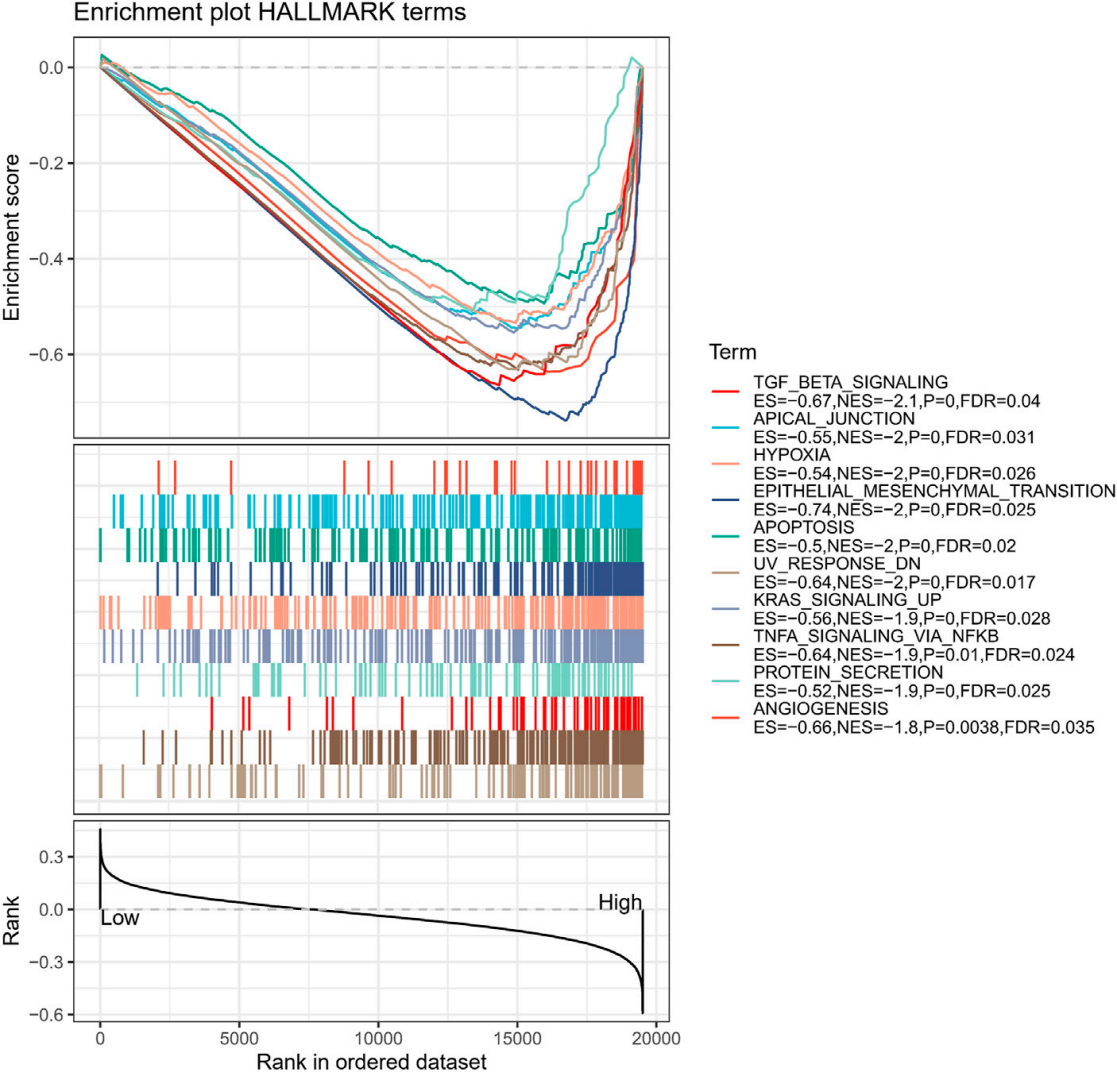
Biological pathway characterisation of APR_Score groups *via* GSEA.

Moreover, to discuss the biological pathway variation in APR_Score groups, we evaluated the markedly enriched pathways in distinct APR_Score groups using GSEA methods *via* h.all.v7.5.1.symbols.gmt signatures. We noted that TGF BETA SIGNALING, APICAL JUNCTION, HYPOXIA, EPITHELIAL MESENCHYMAL TRANSITION, APOPTOSIS, UV RESPONSE DN, KRAS SIGNALING UP, TNFA SIGNALING VIA NFKB, PROTEIN SECRETION, ANGIOGENESIS were markedly enriched in high-APR_Score group (*p* < 0.05, FDR<0.25, Figure 11).

## Discussion

Anoikis is a protective mechanism *via* which the organism self-corrects abnormal disorders in the presence of disorders and damage, and is essential for the normal growth and development process of living organisms (Zhong and Rescorla, 2012). Advanced cancer patients are affected by tumor metastasis, which occurs in large tumors and could result in death (Steeg, 2016). The prerequisite for tumor cells to migrate would be their own resistance to Anoikis, otherwise the self-correcting capacity of the organism could prevent the formation of metastatic lesions (Simpson et al., 2008; Kim et al., 2012). In fact, the existing studies related to the principles of the anoikis mechanism in CC remained limitations.

In this study, we defined three potential subtypes based on anoikis-related lncRNAs in CC that exhibited remarkable prognostic variation as well as immunoreactivity. Notably, AC3 patients exhibited higher ARG scores and angiogenesis scores. Angiogenesis is essential and important in the development of malignant tumor (Viallard and Larrivee, 2017). In contrast, tumor cell anoikis resistance was sufficiently necessary for the formation of new blood vessels and for migration (Kim et al., 2012). The interaction between anoikis and tumor angiogenesis was reported. Gao and colleagues showed that osteosarcoma cells resist through anoikis by activating Src kinase, which in turn activates JNK/ERK/VEGF-A to promote the formation of tumor metastases (Gao et al., 2019). In the present study, we also found marked activation of angiogenesis and NOTCH signaling in the AC3 subtype (Figure 5E). The survival rate of AC3 patients was not satisfactory. This allowed us to speculate that AC3 patients might have a higher probability of postoperative recurrence and higher prognostic risk. However, distal metastases occurred in the majority of cases at the time of diagnosis in CC patients at present (Dereje et al., 2020; Sengayi-Muchengeti et al., 2020; Ryzhov et al., 2021). Accurate determination of disease staging would be crucial for patient treatment modalities and clinical outcomes. Therefore, the ARLs-related subtypes defined in this study might assist M Stage and more accurately calculate the risk of metastasis in CC patients.

Here, we have constructed prognostic risk stratification models for CC based on the expression of ARLs. We explored the potential utility of APR_Score in predicting patient survival and prognosis. In addition, this study also explored the potential function of APR_Score in guiding immunotherapy and chemotherapy. Immunotherapy offered promising opportunities for patients as an emerging option for the rehabilitation of advanced CC patients (Duska et al., 2020; Colombo et al., 2021). Pembrolizumab was approved by the Food and Drug Administration (FDA) as an immunotherapeutic agent for CC (De Felice et al., 2021), however, the current treatment with Pembrolizumab for CC remained limited due to the absence of efficacy biomarkers to determine treatment benefit (Marret et al., 2019). In this study, there were discrepancies in immune cell scores between high- and low-APR_Score patients and in the TIDE scores. We observed higher T cell CD8, T cell CD4 memory resting and TIDE scores in patients with high APR_Score. Tumor cells blocked T cell killing by expression of PD-1/PD-L1, causing immune escape of tumor cells (Pardoll, 2012; Chabanon et al., 2016). Another study concluded that CC cells had a greater tendency of immune escape and were accompanied by increased T cells (Mortezaee, 2020). Moreover, acquisition of anoikis-resistance enhances the abilities of invasiveness, escaping from immune surveillance and therapeutic agents in cancer cells. Fanfone and colleagues have demonstrated that mechanically stressed and anoikis-resistant cancer cells had increased cell motility and escape from killing by natural killer cells (Fanfone et al., 2022), suggesting that anoikis was strongly associated with immune escape of tumor cells. We constructed a prognostic model based on ARLs, and patients with high APR_Score had suboptimal immunotherapy benefit and were more suitable for taking conventional chemotherapy, suggesting that ARLs-based prognostic risk model was a promising predictor in immunotherapy and chemotherapy. Furthermore, the COX model demonstrated that APR_Score was an independent prognostic indicator, and that the nomogram developed in conjunction with APR_Score was a valid prognostic tool. Thus, this finding confirmed the positive utility of ARLs-related signatures in guiding the prognosis and treatment selection, which might provide a theoretical basis for the development of precise and personalized treatment guidelines for CC.

ARLs-related prognostic risk model consisted of AC092614.1, AL158071.3, AC016394.2, LINC02749, MIR100HG, AC079313.1, ATP2A1-AS1. We found that MIR100HG and ATP2A1-AS1 were correlated with multiple cancers and the remaining ARLs were the first identified tumor prognostic markers. Several studies suggested that aberrant expression of MIR100HG was associated with poor clinical outcome and pathological features, and that it was involved in multiple pathways related to tumorigenesis (Wu et al., 2022). aTP2A1-AS1 is a potential prognostic biomarker for CC (Feng et al., 2021). The mechanism of anoikis in CC was not completely elucidated, and the ARLs identified in this study were important for the elucidation of the molecular mechanisms of CC. However, limitations of this study should be equally noted. In other datasets, there are few cervical cancer-related data sets and they lack clinical information (survival time). Due to the difficulty in annotating lncRNAs in other databases, only the TCGA database was used and randomly divided into training set and validation set to construct and validate the risk model in this study. The accuracy of APR_Score should be validated in the future using clinical samples or sequencing data from multiple centers. Finally, the mechanism of 7-ARLs in CC still required further study, which was our follow-up research plan.

## Conclusion

In this study, we defined three molecular subtypes of anoikis-related lncRNAs and generated ARLs-related signatures for assessing the prognosis of patients with CC. The molecular subtypes contributed to a better understanding of the mechanism of CC metastasis and the signatures held potential clinical value in predicting response to therapy.

## Data Availability

The original contributions presented in the study are included in the article/Supplementary Material, further inquiries can be directed to the corresponding author.

## Abbreviations

ARLs, Anoikis-related lncRNAs; APR_Score, Anoikis prognostic risk model; ARGs, Anoikis-related genes; AUC, area under ROC curve; CC, Cervical cancer; FC, fold change; GSVA, Gene set variation analysis; GSEA, Gene set enrichment analysis; HR, hazard ratio; IC50, the half maximal inhibitory concentration; IDE, integrated development environment; K-M, Kaplan-Meier; LASSO, least absolute shrinkage and selection operator; MSigDB, Molecular Signatures Database; OS, overall survival; ROC, receiver operating characteristic analysis; ssGSEA, single-sample gene set enrichment analysis; TCGA, The Cancer Genome Atlas; TIDE, Tumor Immune Dysfunction and Exclusion; TME, Tumor microenvironment.
