# Supplementary figures and images for: Anoikis-related long non-coding RNA signatures to predict prognosis and small molecular drug response in cervical cancer
Source: Front Pharmacol. 2023 Mar 20;14:1135626. doi: 10.3389/fphar.2023.1135626 (PMC10067583; doi:10.3389/fphar.2023.1135626)

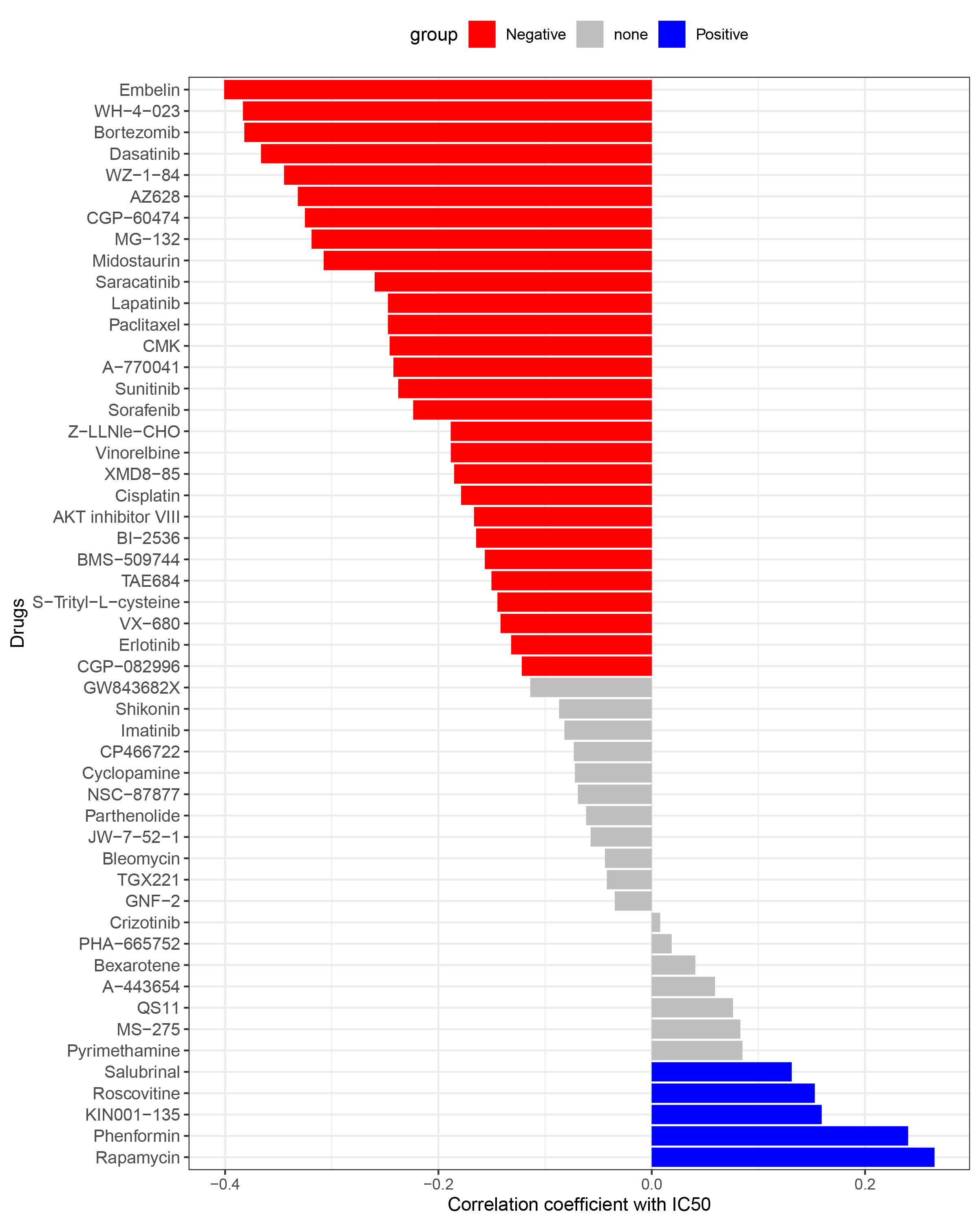

Supplement: Supplementary file 1 [file Image3.JPEG]

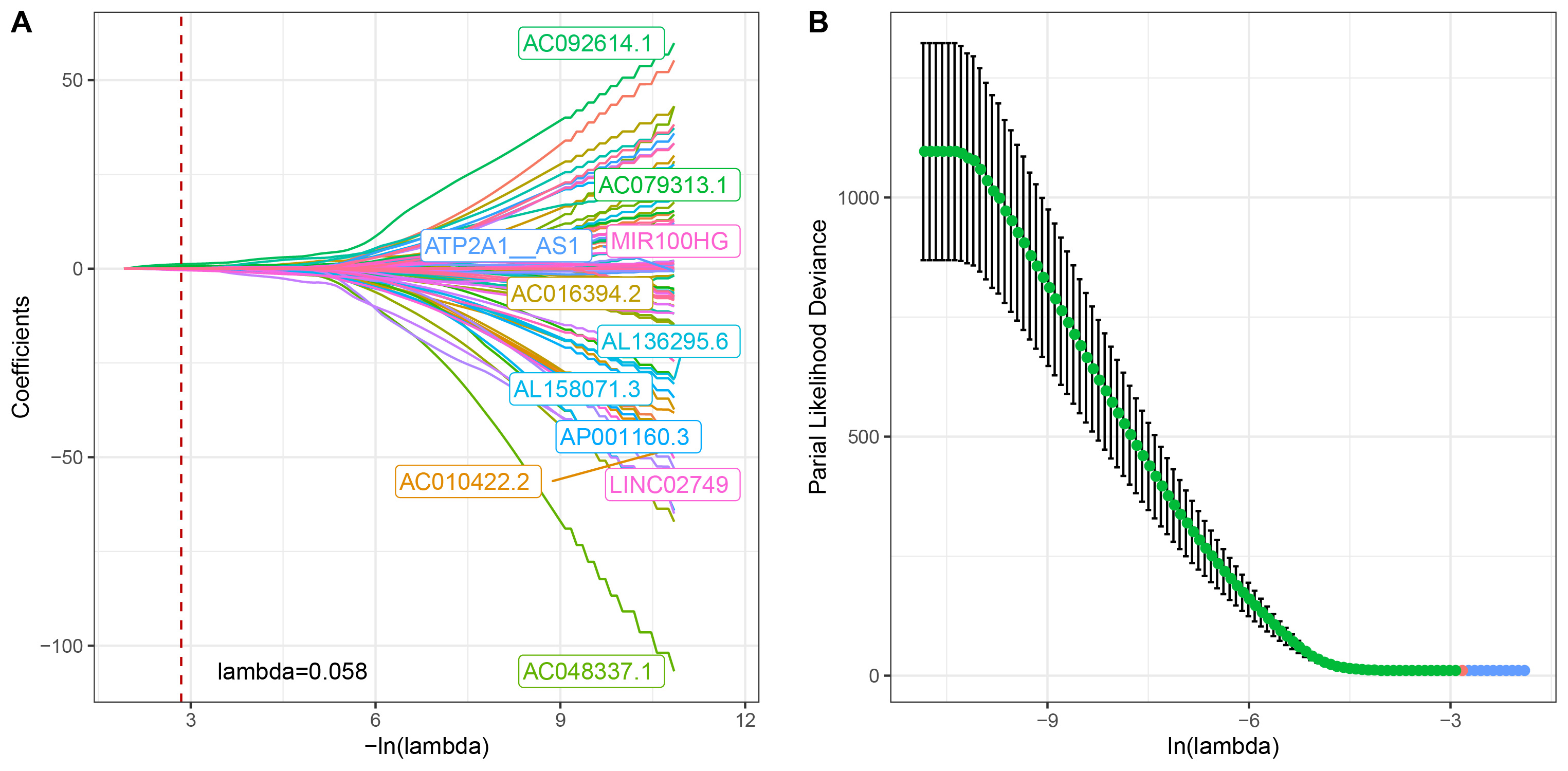

Supplement: Supplementary file 2 [file Image1.JPEG]

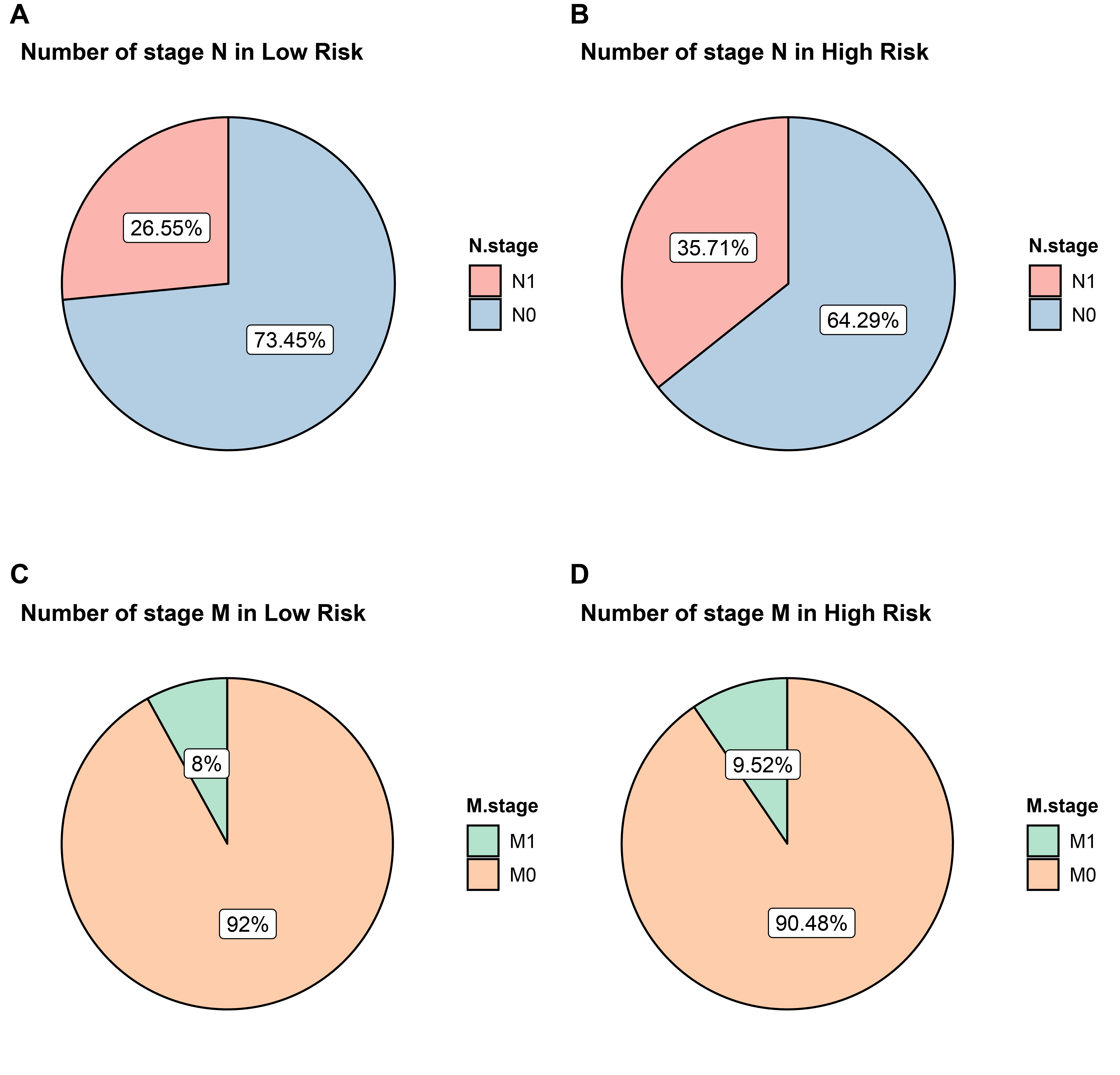

Supplement: Supplementary file 3 [file Image2.JPEG]
