# Supplementary material for: Anoikis-related long non-coding RNA signatures to predict prognosis and small molecular drug response in cervical cancer
Source: Front Pharmacol. 2023 Mar 20;14:1135626. doi: 10.3389/fphar.2023.1135626 (PMC10067583; doi:10.3389/fphar.2023.1135626)
Supplement: Supplementary file 4 [file DataSheet1.docx]

Supplementary Figure 1 LASSO COX model analysis. (A) Variation trajectory of non-zero lncRNAs regression coefficients in LASSO COX model (B) lambda value interval.

Supplementary Figure 2 Differences of clinical characteristics (N satge and M stage) between risk groups in TCGA-CESC cohort. (A-B), Proportions of patients with N stage in low-APR_Score group and high-APR_Score group. (C-D), The number of patients with M stage in low-APR_Score group and high-APR_Score group.

Supplementary Figure 3 Correlation analysis of IC50 values of chemotherapy drugs with APR_Score.
